# USP14 Positively Modulates Head and Neck Squamous Carcinoma Tumorigenesis and Potentiates Heat Shock Pathway through HSF1 Stabilization

**DOI:** 10.3390/cancers15174385

**Published:** 2023-09-01

**Authors:** Jie Wang, Yuandi Xiang, Zhanghong Xie, Mengqi Fan, Shizhen Fang, Huanzhi Wan, Rui Zhao, Feng Zeng, Qingquan Hua

**Affiliations:** 1 Department of Otolaryngology-Head and Neck Surgery, Renmin Hospital of Wuhan University, 238 Jie-Fang Road, Wuhan 430060, China; 2013302180159@whu.edu.cn (J.W.);; 2 Hubei Key Laboratory of Cell Homeostasis, College of Life Sciences, Wuhan University, Wuhan 430072, China

**Keywords:** USP14, deubiquitinase, HSF1, HNSCC, oncogene proteins

## Abstract

**Simple Summary:**

Head and neck squamous cell carcinoma (HNSCC) is the sixth most common cancer in the world, with a heterogeneous characteristic. We firstly systematically screened the critical gene ubiquitin-specific protease 14 (USP14) in HNSCC from the DUB library after building a risk signature. Our data suggested that USP14 regulated heat shock transcription factor 1 (HSF1) stabilization by its deubiquitination and increased its downstream proteins, such as heat shock protein 60 (HSP60), HSP70, and HSP90, to promote HNSCC proliferation and lung metastasis in vivo and in vitro, and the overexpression of HSF1 reversed the inhibitory effect of USP14 depletion in vivo. Forty-five tissue samples from patients with HNSCC cancer were collected to determine the correlation of USP14 and HSF1 expression in the diagnosis of HNSCC, which is a novel mechanism of USP14 as a carcinogenic gene in HNSCC. Our results emphasized that USP14 and its downstream HSF1 have therapeutic potential in targeting HNSCC.

**Abstract:**

The ubiquitin-proteasome system is a pivotal intracellular proteolysis process in posttranslational modification. It regulates multiple cellular processes. Deubiquitinating enzymes (DUBs) are a stabilizer in proteins associated with tumor growth and metastasis. However, the link between DUBs and HNSCC remains incompletely understood. In this study, therefore, we identified USP14 as a tumor proliferation enhancer and a substantially hyperactive deubiquitinase in HNSCC samples, implying a poor prognosis prediction. Silencing USP14 in vitro conspicuously inhibited HNSCC cell proliferation and migration. Consistently, defective USP14 in vivo significantly diminished HNSCC tumor growth and lung metastasis compared to the control group. Luciferase assays indicated that HSF1 was downstream from USP14, and an evaluation of the cellular effects of HSF1 overexpression in USP14-dificient mice tumors showed that elevated HSF1 reversed HNSCC growth and metastasis predominantly through the HSF1-HSP pathway. Mechanistically, USP14 encouraged HSF1 expression by deubiquitinating and stabilizing HSF1, which subsequently orchestrated transcriptional activation in HSP60, HSP70, and HSP90, ultimately leading to HNSCC progression and metastasis. Collectively, we uncovered that hyperactive USP14 contributed to HNSCC tumor growth and lung metastasis by reinforcing HSF1-depedent HSP activation, and our findings provided the insight that targeting USP14 could be a promising prognostic and therapeutic strategy for HSNCC.

## 1. Introduction

HNSCC is the sixth most common cancer in the world [1], and its 5-year survival rate is 50% [2]. Currently, the primary treatment is surgery combined with radiotherapy, chemotherapy, or molecular-targeted therapy [3]. Previous molecular and cytogenetic studies have already suggested that mutations or the abnormal expression of various oncogenes and tumor suppressors promote the malignant phenotype of HNSCC. Despite advances in both clinical and basic research, there has not been much benefit for patients with HNSCC. Therefore, an in-depth study at the molecular level is essential to select the most suitable treatment for high-risk and low-risk patients and to provide novel ideas and methods for the treatment of HNSCC.

The ubiquitin-proteasome system is a main pathway for endogenous protein degradation; in addition to the lysosomal pathway, it is of great significance in cellular homeostasis and processes [4,5]. Deubiquitinating enzymes (DUBs) are proteases that reverse protein ubiquitination [6], which plays an important role in the development of malignant tumors. It was previously shown that USP14 can stabilize JNK to promote colorectal cancer proliferation [7] and can catalyze the deubiquitination of TAZ to drive pancreatic ductal adenocarcinoma progression [8]. Inhibiting USP14 could trigger autophagy via ER stress-mediated UPR in lung cancer [9]; moreover, USP14 deubiquitinates CDK1 in breast cancer to promote its cell cycle [10]. However, the role of USP14 and its downstream in HNSCC remains unclear.

Heat shock response is an acute and transient process occurring when normal human cells are exposed to changes in the body’s internal environment, such as temperature, inflammation, or hypoxia [11,12,13]. Malignant tumors are the epitome of pathology and are a result of chronic toxic protein stress, which inevitably interferes with malignant transformation and cellular homeostasis. Heat shock response can stabilize cancer cells by protecting protein homeostasis; strengthening the drug resistance of cancer cells [14]; and promoting the migration, invasion, metastasis, and proliferation of tumor cells [15,16,17]. HSF1 is a major trans-activator of the adaptive thermogenesis pathway, especially heat shock response. HSF1 generally binds to five nGAAn polymers of the heat shock element (HSE) [18] and subsequently induces the transcription of downstream heat shock proteins (HSPs), such as HSP60, HSP70, and HSP90, which then proceed to induce the proteolytic degradation of damaged proteins, and these HSPs provide critical stress relief and exhibit metastasis promotion roles in cancers [19,20,21,22,23]. In cancer cells, heat shock treatment (43 °C for 2 h) leads to a rapid increase in HSF1 binding to HSP promoters; then, HSP gene transcription increases sharply when the state of HSF1 in tumor cells is constitutively activated [16,24,25]. It has been found that HSF1 is mainly regulated by posttranslational modification in cells, such as phosphorylation, ubiquitination, and acetylation [26,27,28], but the deubiquitylation regulation of HSF1 is poorly understood.

In the present study, we found that USP14 was upregulated in HNSCC tissues and was associated with HNSCC patients’ poor prognosis. We also demonstrated that USP14 increased HNSCC cell proliferation and metastasis. USP14 acted as a positive regulator of HSF1 by stabilizing and deubiquitinating the HSF1 protein. Hence, USP14 could be a potential therapeutic target for HNSCC patients.

## 2. Materials and Methods

### 2.1. Cell Culture and Materials

The HNSCC cell lines Cal-27 were purchased from the Wuhan University cell bank (Wuhan, China). TU686 and SCC9 were purchased from Hunan Fenghui Biotechnology Co., Ltd. (Hunan, China). SNU1076, TU177, and SCC7 were donated by the First Affiliated Hospital of Sun Yat-sen University. DMEM (HyClone, Logan, UT, USA), 10% FBS (PAN, Aidenbach, Germany), and 1% penicillin-streptomycin (10,000 U/mL penicillin and 10,000 g/mL streptomycin; Life Technologies, Carlsbad, CA, USA) were utilized for maintenance cultures for these cell lines. The cells were kept in a humidified incubator at 37 °C in 5% CO_2_.

### 2.2. Data Acquisition

The RNA sequencing datasets were downloaded from The Cancer Genome Atlas (TCGA) database, and the associated clinical data were downloaded (https://portal.gdc.cancer.gov/ (accessed on 2 February 2022)) and analyzed using the R package TCGA-Assembler. The GSE41613 dataset, including 97 matching pairs of HNSCC tumor and adjacent nontumor tissues, was downloaded from Gene Expression Omnibus (GEO). In total, 126 deubiquitinating-associated genes (DAGs) were downloaded from the GSEA database. All data were processed with R software.

### 2.3. HNSCC Tissue Microarray and Samples from Individuals with HNSCC

Forty-five HNSCC cancer and corresponding adjacent tissue chips were purchased from Whu Iwill Biological Technology Co., Ltd. (IWLT-N-90LS51) to explore the expression of USP14 and HSF1 in tissue samples by using IHC staining. Nine pairs of HNSCC and adjacent normal tissues were taken from the patients at the moment of surgical resection, all of which were obtained from the Department of Otolaryngology-Head and Neck Surgery, Renmin Hospital of Wuhan University. None of the patients had received radiotherapy or chemotherapy before surgery. Through the examination of medical records and pathological reports, information about the characteristics of the patients and their tumors was obtained. The ethics committee of Renmin Hospital at Wuhan University approved the informed consent form after reviewing it (certificate number WDRY2021-KS077).

### 2.4. Establishment of the DAG-Related Risk Signature

To create an optimal prognostic signature for HNSCC samples and select these potential biomarkers, 126 DAGs were acquired from the GSEA database, and a LASSO Cox regression analysis was performed using the R glmnet package. The signature successfully achieved shrinkage and successfully chose variables using the LASSO regression penalty, and the optimal value was identified by 10 cross-validations with the lowest likelihood of divergence. The prognostic expression of DAGs of every patient and the related coefficient were calculated, and the DAG risk score was calculated as follows: risk score = $\sum_{k=1}^{n}coef({DAG}_{k})*expr\left({DAG}_{k}\right)$, where coef (DAGn) presents the LASSO regression coefficient of the gene n, and expr (DAGn) presents its corresponding expression of DAGs.

### 2.5. Plasmids and Reagents

Human USP14 WT cDNA and USP14 C114A mutant cDNA were cloned and amplified from the TU686 cDNA library and subsequently subcloned into the pCDNA3.0 plasmid, which contains the 3×Flag tag. Human HSF1 WT was also amplified from the TU686 cDNA library and subcloned into the pCDNA3.0 plasmid, which contains the 3×HA tag. The USP14 primary antibodies used for immunoblotting and immunohistochemical (IHC) assays were as follows: rabbit polyclonal anti-HSF1 antibody (16107-1-AP, Protein tech; (Wuhan, China), 12972S, Cell Signaling Technology (CST), rabbit polyclonal anti-USP14 antibody (14517-1-AP, Protein tech; 11931S, CST), rabbit polyclonal anti-HSP70 antibody (10995-1-AP, Protein tech), rabbit polyclonal anti-HSP90 antibody (13171-1-AP, Protein tech), and FLAG (66008-4-Ig, Protein tech) (Rosemont, IL, USA). Antibodies against HA (M180-3) were obtained from MBL Beijing Biotech Co., Ltd. (Beijing, China). The proteasome inhibitor MG-132 (HY-13259) and the protein synthesis inhibitor cycloheximide (HY-12320) were obtained from MCE, China. The USP14 shRNAs were ligated into PLV/U6 (Addgene), and the USP14 shRNA plasmids (mouse and human) were purchased from Miao Ling Plasmid, China.

### 2.6. Real-Time RT-PCR

RNA extraction from HNSCC cells and primary cancer tissues was performed using RNAiso Plus (TaKaRa Biomedical Technology, 9108, Shiga, Japan) according to the manufacturer’s instructions. Subsequently, a microgram of RNA was reverse-transcribed using the RevertAid first-strand cDNA synthesis kit (Thermo, #K1622. Waltham, MA, USA) after quantifying mRNA by quantitative PCR. RT-PCR was then performed using SYBR Green qPCR Master Mix (KUBO, q225mix, Beijing, China) on a Roche LightCycler^®^ 480II real-time PCR system (Roche, Basel, Switzerland). All real-time PCR values were normalized to β-actin mRNA expression. The primers used in the study are presented in Supplementary Table S1.

### 2.7. Fluorescence Microscopy

To evaluate the subcellular localization of USP14 and HSF1, the Cal-27, TU686, and TU177 cell lines were fixed in 4% paraformaldehyde and then permeabilized in 0.5% Triton X-100. After blocking in blocking buffer (5% bovine serum albumin in PBS) for 1 h, membranes were incubated with a primary polyclonal antibody USP14 polyclonal antibody (1:25, 14517-1-AP, Proteintech) or anti-HSF1 (1:50, 51034-1-AP, Protein tech, USA) overnight at 4 °C, followed by exposure to a secondary antibody tagged with Alexa 488 or Alexa 568 (Molecular Probes, Invitrogen, Waltham, MA, USA). The nuclei were stained with DAPI (Sigma-Aldrich, Shanghai, China), and confocal fluorescence images were captured under a fluorescence microscope (Leica, Wetzlar, Germany).

### 2.8. CCK8 Assay

HNSCC cells were seeded at a density of 1000 cells per well in 96-well plates in triplicate. CCK-8 solution (diluted in 1/10 DMEM) was then added to each well, and the cells were incubated at 37 °C for 1 h to detect viable cells. Subsequently, the absorbance of each well was detected at a wavelength of 450 nm.

### 2.9. Colony Formation

To test the long-term proliferation capacity of HNSCC cells, the cells were seeded in 6-well plates at a density of 1000 cells/well, and the incubation period was 14 days at 37 °C. After fixing the cells with 4% formaldehyde, the plates were subsequently stained with 0.5% crystal violet solution. The colonies were counted using ImageJ v 1.43 software after progressively staining the plates.

### 2.10. Cell Migration

The transwell chamber system was used to evaluate the HNSCC cell migration ability after gene editing. We cultured 5 × 10^4^ resuspended HNSCC cells in serum-free DMEM for 24 h at 37 °C before seeding cells in the Transwell chamber. Subsequently, the cells were switched to a serum-free medium, and DMEM with 15% FBS was added to the bottom chamber. After 48 h, cells that had migrated to the bottom surface of the membrane were preserved with formaldehyde, dyed with 0.5% crystal violet, and quantified using ImageJ.

### 2.11. Wound-Healing Assay

Wound-healing assays were applied to assess the migration ability of HNSCC cells after gene editing. Each group of cells was seeded in 6-well plates and incubated in 10% FBS medium at 37 °C until they reached 80% confluence. Subsequently, a 10-µL pipette tip was used to scratch a line along the cell monolayer, and the medium was changed to a serum-free medium. After incubation for 12 h and 24 h at 37 °C, the average scratch closure was observed by a light microscope.

### 2.12. Dual-Luciferase Reporter Assay

HEK 293T cells were seeded in 24-well plates and transfected with the indicated plasmids (containing 16 signaling pathways: ATF2/3/4, NRF2, GATA, HSF1, STAT3, FOXO3, Myc, HIF-1, ER stress, PPAR, KLF4, HNF4, NF-kB, P53, Pax6, and SMAD2/3/4). pRL-TK (10 ng), pGL4-reporter plasmids (100 ng), and pHAGE/pHAGE-USP14 plasmids (400 ng) for 36 h. The reporter assays were then tested using a dual-specific luciferase assay kit (Promega, Madison, WI, USA).

### 2.13. Flow Cytometry

To determine apoptosis, the cells were treated with propidium iodide (PI) and Annexin V-fluorescein isothiocyanate (Annexin V-FITC), according to the manufacturer’s instructions (BD Biosciences, San Jose, CA, USA). To test the cell cycle, the cells were washed with PBS after being fixed with 70% ethanol overnight and collected in centrifuge tubes. The cell cycle alterations between the control and treated groups were tested by a Cell Cycle Detection Kit (Biyuntian, Shanghai, China). Then, the cells in these two assays were both analyzed by a flow cytometer (Beckman Coulter, Brea, CA, USA), and gates were established in reference to unstained controls stained and fluorescence-minus-one controls.

### 2.14. Coimmunoprecipitation Assays

HEK293T cells were transfected with the indicated plasmids for 36 h and harvested in NP-40 lysis buffer supplemented with a protease inhibitor cocktail (Roche, Basel, Switzerland). Immunoprecipitation was performed with 2 μg anti-Flag or anti-HA antibodies overnight (MBL International, Woburn, MA, USA), and protein A/G magnetic beads were added to the lysates (Thermo Fisher Scientific, Waltham, MA, USA) for 4 h. The immunoprecipitated proteins were eluted and separated using SDS-PAGE and visualized in the ChemiDocTM MP Imaging System (Bio-Rad, Hercules, CA, USA).

### 2.15. Lung Metastasis and Tumor Xenograft Model

NOD/SCID nude mice (6 weeks old) and BALB/c-nude mice (8 weeks old) were purchased from Charles River (Beijing, China) and housed under specific pathogen-free conditions. There were three types of mice model we constructed, and 2 × 10^6^ cells were, respectively, orthotopically injected into BALB/c-nude mice, subcutaneously injected into NOD/SCID nude mice, and also intravenously injected into NOD/SCID nude mice. Subcutaneous tumor volumes of NOD/SCID nude mice were measured every 3 days and calculated according to the formula: V = lw^2^/2 (l = tumor length, w = tumor width). After euthanasia of the mice, the subcutaneous tumors were weighed, and the tumors, lungs, and tongues of mice were separated for subsequent HE staining, IHC, and Western blotting assays. The Ethics Committee on the Care and Use of Laboratory Animals of Wuhan University approved the protocol used in animal experiments (permit number: IACUC2018055).

### 2.16. Deubiquitination Assay

Plasmids encoding Myc-ubiquitin, Flag-USP14, and HA-HSF1 were transfected into HEK 293T cells. After 30 h, 10 μM MG132 was added to 293T cells for 8 h. Anti-HA was incubated with protein A/G in agarose cellular extracts overnight for 12 h; thereafter, the ubiquitinated HSF1 levels were evaluated using immunoprecipitation and Western blotting assays.

### 2.17. Luciferase Live Imaging

Female NOD/SCID mice at the age of 6 weeks were individually administered with 3 × 10^5^ specified cells via the tail vein. After a period of 21 days, the mice were subjected to anesthesia using isoflurane, followed by an intraperitoneal injection of 200 µL D-luciferin potassium salt (150 mg/kg in PBS, Beyotime, ST196) 10 min prior to live imaging. The PEIVIS Spectrum in vivo imaging system was employed to capture images, which were subsequently analyzed using Living image 4.0 software. All lung metastasis tumors were surgically removed and preserved in 10% formaldehyde for histological examination.

### 2.18. Statistical Analysis

Data were analyzed by the mean ± standard deviation (SD) at least three times. Error bars were used to evaluate the data from biological studies that were performed in triplicate. Two groups of data were compared and analyzed by an unpaired two-tailed Student’s *t*-test, and multiple groups of data were compared by one-way ANOVA. CCK8 was analyzed by two-way ANOVA. *p*-values < 0.05 were considered statistically significant.

## 3. Results

### 3.1. Identification of USP14 as a Potent Promoter of HNSCC Patient Survival

**Figure 1:**
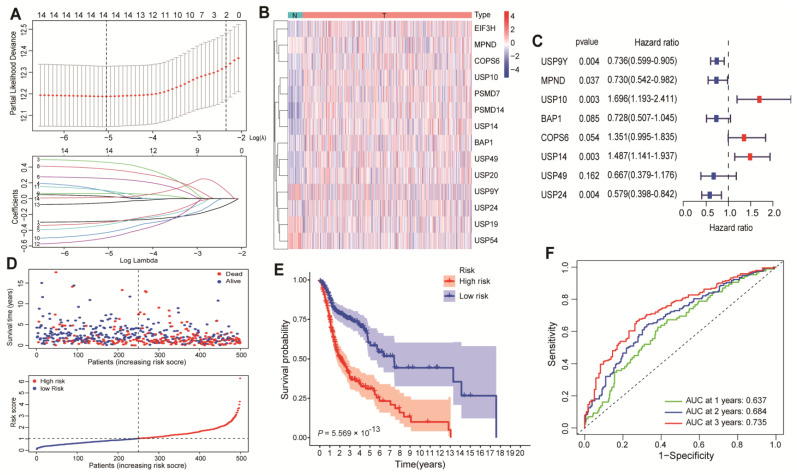
Extraction of the prognostic signature of DAGs in HNSCC and identification of an optimal biomarker for HNSCC. (**A**) The 10-fold cross-validation for the optimal variable selection in the LASSO coefficient profiles of 14 DAGs. (**B**) Heatmap showing the fourteen differentially expressed prognostic genes that were extracted by Lasso regression analysis. (**C**) Eight prognostic genes were extracted by multivariate Cox regression analysis. (**D**) The distribution of the DAG model-based risk score and overall survival time. The dotted line presents the cut-off point for the middle risk score, by which patients were separated into low-risk and high-risk groups. (**E**) Kaplan-Meier survival curves of overall survival showing the survival time and survival status between the low- and high-risk groups. (**F**) ROC curve showing the 1-year, 2-year, and 3-year AUCs of the DAG signature to predict the prognosis of HNSCC.

The mRNA expression data of 542 HNSCC patients were extracted from TCGA, and another 97 HNSCC patients were downloaded from GSE41613 (GEO databases). Deubiquitinating-associated genes (DAGs) were obtained from GSEA for subsequent analysis. Ninety-three differentially expressed DAGs between normal patients and HNSCC patients among TCGA and GSE41613 overlapped (Figure S1A). The univariate Cox regression analysis was used to screen 66 DAGs that were related to the overall survival (OS) of HNSCC patients (Figure S1B), as shown in the volcano plot in Figure S1C. Then, Lasso Cox regression was performed, and 14 DAGs were extracted when the optimal value of λ showed the minimum likelihood of deviation (Figure 1A,B). The multivariate Cox regression analysis proceeded to choose eight optimal DAGs for the HNSCC biomarkers (Figure 1C), and a signature was built to predict the OS in patients. The risk score was calculated according to the previous formula, and each patient had a risk score. All patients were equally divided into low- and high-risk groups according to this signature (Figure 1D), and patients with low-risk scores had better overall survival than those with high-risk scores in terms of the formula (Figure 1E). The areas under the curve (AUCs) of the predictive precision of the signature were 0.637 at 1 year, 0.684 at 2 years, and 0.735 at 3 years, according to a time-dependent receiver operating characteristic (ROC) analysis (Figure 1F). Among all the identified genes, we selected the following oncogenes for in vitro functional screening: USP14, USP10, and COPS6. Specifically, COPS6 has been reported to play a carcinogenic role in HNSCC [29], and prior experiments showed that USP14-deficient HNSCC cells had a significantly reduced proliferation rate and cell migration ability compared to USP10 ablation. Hence, we focused on USP14 for further study.

### 3.2. USP14 Is Highly Expressed in HNSCC Tumors and Predicts Poor Clinical Outcomes in HNSCC Patients

**Figure 2:**
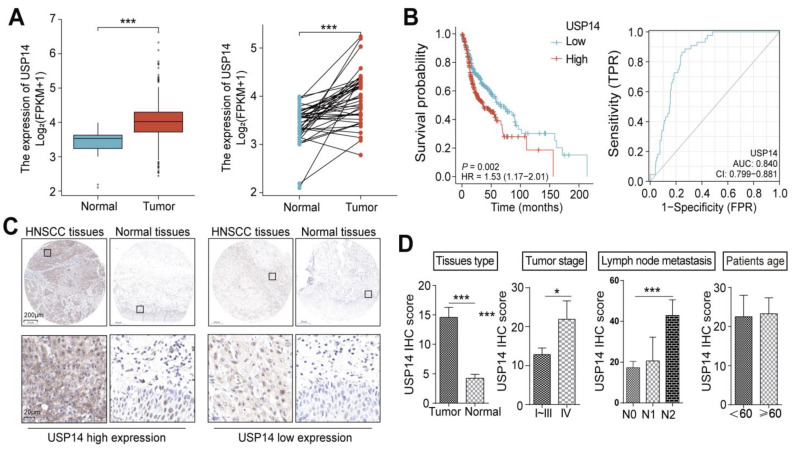
USP14 is highly expressed in HNSCC tumors, and its upregulation is associated with a poor prognosis in HNSCC patients. (**A**) The USP14 mRNA levels were evaluated in HNSCC tissues (T) compared to paired or random precancerous tissues (N) from the TCGA database. (**B**) Kaplan-Meier overall survival curves of USP14 high and low expression in the prognosis of HNSCC patients in the TCAG database. The ROC curve showed that USP14 had obvious prognostic value in HNSCC (AUC = 0.840). (**C**,**D**) Representative images and the quantification of USP14 IHC staining in HNSCC cancer and normal tissues derived from patients, and among all the clinical characteristics, the USP14 IHC score was significantly associated with patients’ tissue type, tumor stage, and lymph node metastasis. * Indicates *p* < 0.05, and *** indicates *p* < 0.0001.

To determine the correlation between USP14 expression and HNSCC patients’ clinical characteristics, we evaluated USP14 mRNA expression by analyzing TCGA HNSCC cancer datasets. The USP14 mRNA levels were significantly elevated in HNSCC tissues compared with normal tissues (Figure 2A). Furthermore, the Kaplan-Meier plotter analysis (https://kmplot.com/analysis (accessed on 1 February 2022)) revealed that patients with higher expression levels of USP14 had poorer clinical OS (Figure 2B). ROC curve analysis showed that the AUC of USP14 was 0.840, indicating that the prognostic precision of USP14 in patients was relatively high. IHC assays with 43 paired HNSCC patient tissue chips revealed a higher expression of USP14 in HNSCC tissues than in para-carcinoma tissues (Figure 2C). In addition, the expression level of USP14 was associated with the clinical stage and lymph node metastasis of HNSCC patients (Figure 2D). The association between USP14 expression and the clinicopathological features of HNSCC patients is shown in Supplementary Table S2. Collectively, these results indicated that USP14 might be an oncogene in HNSCC.

### 3.3. Knockdown of USP14 Weakened HNSCC Cell Proliferation and Metastasis

**Figure 3:**
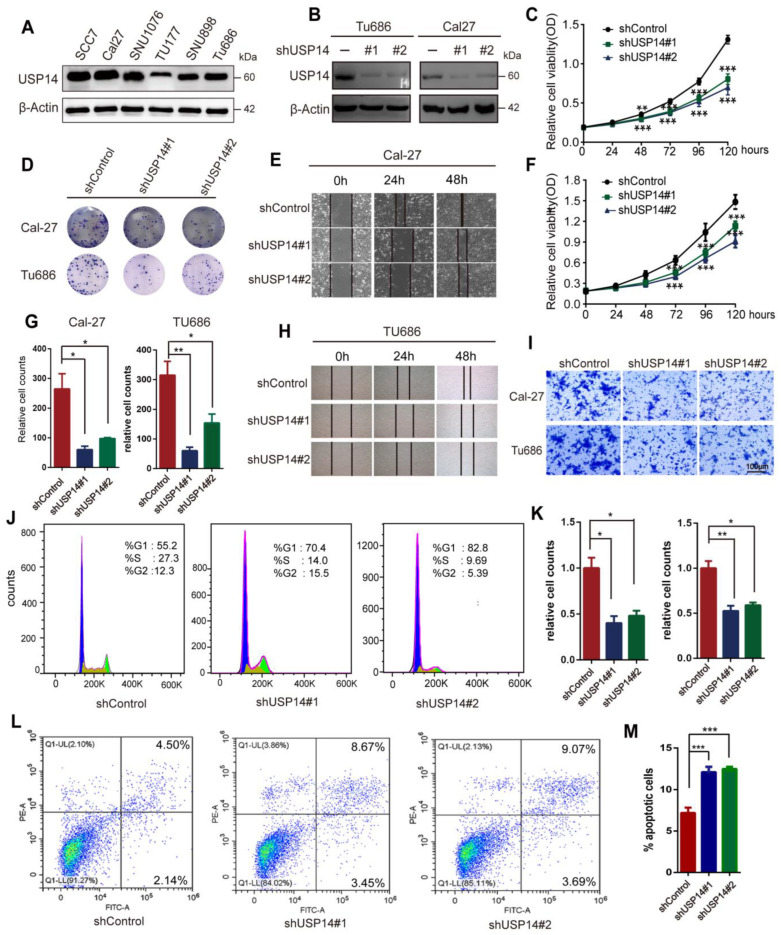
The depletion of USP14 decreases HNSCC cell proliferation and migration in vitro. (**A**) USP14 protein expression in HNSCC cell lines. (**B**) Lentiviruses were used to knock down USP14 in Cal-27 and TU686 cell lines with shUSP14RNA#1 and shUSP14RNA#2. (**C**,**F**) CCK8 assays were used to test the proliferation ability of Cal-27 and TU686 cells with USP14 knockdown. (**D**,**G**) Colony formation assays were performed to examine the cell formation ability of USP14-deficient Cal-27 and TU686 cells. (**E**,**H**) Wound-healing assays were conducted to test the cell migration ability after the depletion of USP14 in Cal-27 and TU686 cells. (**I**,**K**) The cell migration ability was assessed by Transwell migration assays in Cal-27 and TU686 cells. ImageJ software was used to quantify the number of cells. (**J**) A cell cycle analysis was used to detect the effect of USP14 on the Cal-27 cell cycle and was analyzed by FlowJo V10 software. (**L**,**M**) The apoptosis rate of Cal-27 cells was analyzed by flow cytometry. Data are analyzed by the mean ± SD at least three times. * Indicates *p* < 0.05, ** indicates *p* < 0.001, and *** indicates *p* < 0.0001.

To verify our hypothesis that USP14 might function as an oncogene in HNSCC cells, we evaluated USP14 expression levels in six distinct HNSCC cell lines, SCC7, Cal-27, SNU1076, SNU899, TU177, and TU686. The results showed that USP14 expression was relatively higher in Cal-27, SCC7, and TU686 cells and lower in TU177 cells (Figure 3A). Thus, we used TU686 and CAL-27 cells to construct USP14 knockdown cell lines and used TU177 cells to construct USP14 and USP14 C114A (Cys114 of USP14 was replaced with an Ala residue to acquire a Ub hydrolase-deficient mutant) overexpression cell lines, respectively. The USP14-depleted cell lines using two independent shRNA were validated by Western blotting (Figure 3B). The CCK8 assay demonstrated that USP14 depletion remarkably impaired Cal-27 and TU686 cell proliferation (Figure 3C,F). Clonogenic assays indicated that USP14 depletion sharply decreased the HNSCC cell colony formation capacity (Figure 3D,G). The wound-healing assay confirmed that USP14 depletion decreased the migration ability of HNSCC cells (Figure 3E,H), and the quantitation is shown in Figure S2A,B. Transwell assays indicated that USP14 depletion significantly reduced the migration ability of Cal-27 and TU686 cells (Figure 3I,K). Furthermore, USP14 depletion caused cell cycle arrest in the G1 phase (Figure 3J) and a higher apoptotic rate compared to control Cal-27 and TU686 cells (Figure 3L,M). Taken together, these data revealed that USP14 depletion inhibited HNSCC proliferation and metastasis and increased HNSCC apoptosis in vitro.

### 3.4. Overexpression of USP14 Promoted HNSCC Cell Proliferation and Metastasis

**Figure 4:**
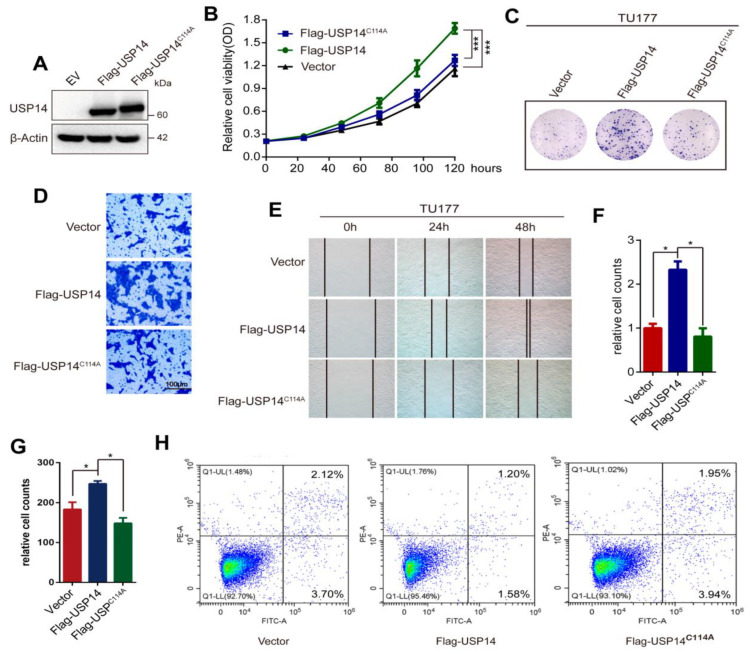
USP14 overexpression increases HNSCC cell proliferation and migration. (**A**) Western blotting showed that Flag-USP14 and Flag-USP14 C114A were successfully transfected into TU177 cells. (**B**) The CCK8 assay was used to examine the proliferation of TU177 cells transfected with USP14, Flag-USP14 C114A, or an empty vector (EV). (**C**,**F**) A colony formation assay was employed to test the effect on the TU177 cell formation ability after USP14, EV, or Flag-USP14 C114A treatment. (**D**,**G**) Transwell migration assays were used to test the migration ability of TU177 cells, and ImageJ software was used to quantify the number of cells. (**E**) Wound-healing assays were conducted to examine the cell migration ability of TU177 cells after USP14, EV, or Flag-USP14 C114A treatment. (**H**) The apoptosis rate of TU177 cells was analyzed by flow cytometry after USP14, Flag-USP14 C114A, or EV transfection. Data are analyzed by the mean ± SD at least three times. * Indicates *p* < 0.05, and *** indicates *p* < 0.0001.

We also transfected Flag-USP14 and Flag-USP14 C114A (USP14 catalytically inactive variant) into the TU177 cell line to verify the oncogenic function of USP14. Western blotting indicated that Flag-USP14 and Flag-USP14 C114A were successfully transfected into TU177 cells (Figure 4A). The CCK8 assay showed that the overexpression of USP14, but not Flag-USP14 C114A, promoted the proliferation of TU177 cells (Figure 4B). Clonogenesis assays showed that the overexpression of USP14 increased colony formation in HNSCC cells, whereas mutant USP14 did not have this ability (Figure 4C,F). Transwell assays verified that the migration ability was increased after USP14 overexpression in TU177 cells (Figure 4D,G). Wound-healing assays showed that the overexpression of USP14 promoted the HNSCC cell migration capacity, while the USP14 mutant type did not (Figure 4E), and the quantitation is shown in Figure S2C. Furthermore, the flow cytometric analysis revealed that the overexpression of USP14 in TU177 cells reduced cell apoptosis compared to the control or Flag-USP14 C114A (Figure 4H). Collectively, these data revealed that the overexpression of USP14 promoted HNSCC proliferation and metastasis and decreased cell apoptosis in vitro.

### 3.5. USP14 Regulates the HSF1 Signaling Cascade

**Figure 5:**
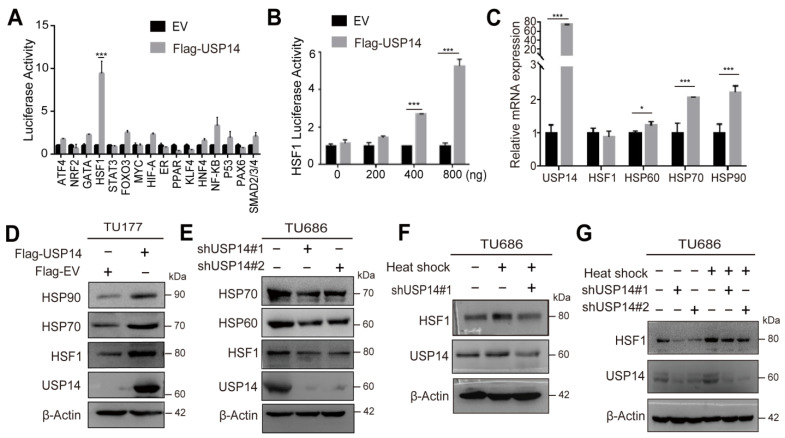
Overexpression of USP14 leads to increased HSF1 signaling. (**A**) Luciferase assays revealed that the most active pathway after USP14 transfection was the HSF1 signaling pathway. (**B**) The luciferase reporter assay showed the cell luciferase activity after HEK 293T exogenously transfected USP14 and EV data from three separate biological replicates. (**C**) Real-time PCR was performed to evaluate the variation in the downstream HSF1 mRNA levels, including HSP60, HSP70, and HSP90, in the Flag-USP14-overexpressing TU686 cell line. (**D**) The TU177 cell line was successfully exogenously transfected with USP14, and immunoblotting was employed to assess the HSF1 expression and its downstream proteins. (**E**) Immunoblotting showed the USP14 protein, HSF1, and its downstream protein expression in USP14-deficient Cal-27 cells. (**F**) TU686 and USP14-deficient TU686 cells were treated with heat shock treatment (42 °C for 1 h), and USP14 and HSF1 proteins were analyzed by immunoblotting. (**G**) TU686 cells were transfected with shUSP14RNA#1, shUSP14RNA#2, or shControl following heat shock treatment (42 °C for 1 h) or at normal temperature, and then, the protein levels of HSF1 and USP14 were tested by immunoblotting. Data are analyzed by the mean ± SD at least three times. * Indicates *p* < 0.05, and *** indicates *p* < 0.0001.

To reveal the underlying mechanism of USP14 in HNSCC cell proliferation and metastasis, a luciferase assay was used to screen for key signaling pathways affected by USP14. Among 16 cancer-related pathways, a high expression of USP14 was found to mainly activate the HSF1 pathway (Figure 5A). Accordingly, a gradient overexpression of USP14 could activate the HSF1 pathway in a dose-dependent manner (Figure 5B). Furthermore, the overexpression of USP14 also increased the mRNA levels of genes targeted by HSF1, such as HSP60, HSP70, and HSP90, but not the HSF1 mRNA levels (Figure 5C), while exogenous USP14 increased the protein levels of both HSF1 and its target proteins (Figure 5D), suggesting that USP14 modified HSF1 at the protein level. The knockdown of USP14 decreased the expression of HSF1 and its downstream proteins (Figure 5E). Furthermore, the expression of HSF1 was elevated in HNSCC cells after incubation under heat shock at 42 °C for 1 h, which was abrogated after the knockdown of USP14 (Figure 5F). Given that the heat shock transcription factor HSF1 is activated rapidly in response to heat shock, we found that USP14 deficiency could compensate for increased HSF1 protein expression after the heat shock response (Figure 5G). Meanwhile, we downloaded the mRNA sequencing data of 502 HNSCC patients from the TCGA database, and the median value of USP14 expression was used to divide patients into USP14 high and low expression groups. It revealed that patients with high USP14 expression exhibited an upregulation of HSP-associated proteins (Figure S3A). The GEO database (GSE39366) also identified that the adaptive thermogenesis pathway was enriched in patients with high USP14 expression, and HSF1 was the transcription factor most involved in this pathway (Figure S3B). Collectively, these data suggest that USP14 modulated the HSF1 signaling cascade.

### 3.6. USP14 Stabilizes the HSF1 Protein by Deubiquitylation of HSF1

**Figure 6:**
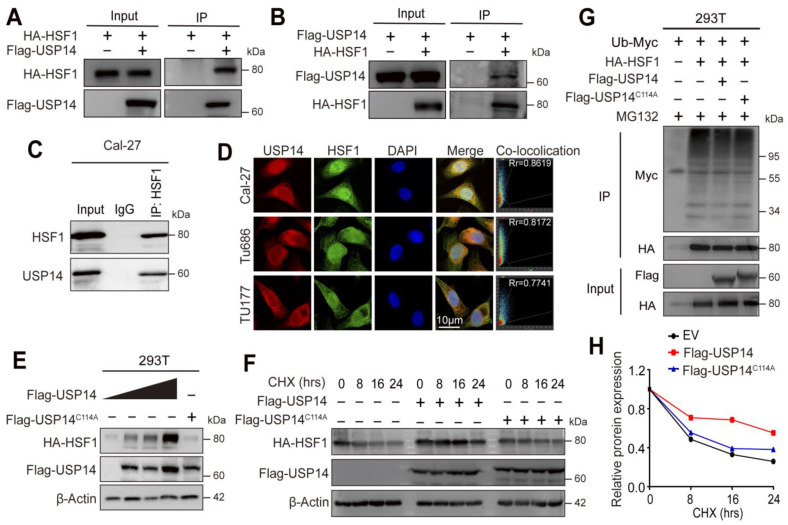
USP14 interacts with HSF1 to modulate HSF1 deubiquitylation. (**A**,**B**) HEK293T cells were co-transfected with exogenous Flag-USP14, HA-HSF1, or the vector, and HA or Flag Sepharose was incubated with the cell extracts. Immunoprecipitates were centrifuged and tested with anti-HA or anti-Flag antibodies by immunoblotting. (**C**) Endogenous USP14 was immunoprecipitated with HSF1 antibodies and tested by Western blotting in Cal-27 cell lines. (**D**) The endogenous colocalization of HSF1 and USP14 in Cal-27, TU686, and TU177 cells was assessed by immunofluorescence. Cells were fixed and labeled with USP14 antibody (red) and HSF1 antibody (green). The nuclei were dyed with DAPI (blue). Scale bar: 10 μm. (**E**) HEK293T cells were exogenously transfected with 0 ng, 500 ng, 1000 ng, 1500 ng USP14-Flag plasmids, and 1000 ng USP14 mutation Flag plasmids, as well as 1000 ng HSF1-HA plasmids, and the stabilization of HA-HSF1 was tested by immunoblotting. (**F**,**H**) HEK293T cells were transfected with the vector or Flag-USP14 or Flag-USP14C114A for 36 h following 100 mg/mL cycloheximide (CHX) treatment for the indicated time periods, and then, cell extracts were collected and tested by immunoblotting, and the protein levels were analyzed. (**G**) HEK293T cells were transfected with the indicated plasmids for 48 h and then treated with 20 μM MG132 for 6 h. Cell extracts were digested under denaturing conditions. HA Sepharose beads were used to collect substrate proteins, and the centrifuged deposit was tested by immunoblotting with the indicated antibodies.

To investigate whether USP14 could interact with HSF1 at the protein level, coimmunoprecipitation (CoIP) assays were performed to explore the possibility of exogenous interactions between USP14 and HSF1 (Figure 6A,B). The results showed that HSF1 physically interacted with USP14 in 293T cells. Furthermore, we confirmed the endogenic interactions of HSF1 and USP14 in Cal-27 cells, as shown in Figure 6C. Next, an immunofluorescence assay was utilized to assess the cellular localization of USP14 and HSF1 (Figure 6D) in Cal-27, TU686, and TU177 cells, and the results showed that USP14 and HSF1 could be colocalized in these cells. To further validate that HSF1 stability was regulated by USP14, HSF1-HA and USP14-Flag (0, 500 ng, 1000 ng, and 1500 ng) or USP14C114A-Flag (1000 ng) were co-transfected into 293T cells. The Western blot analysis showed that USP14 increased the HSF1 protein levels in a dose-dependent manner, while the USP14C114A mutant did not alter the HSF1 protein levels (Figure 6E). To further confirm that USP14 allows the stable accumulation of HSF1 in HNSCC cancer, a cycloheximide chase (CHX) analysis was performed on HSF1 degradation, and we discovered that the HSF1 protein half-life was significantly enhanced when USP14 was overexpressed compared to when the USP14C114A mutant was overexpressed (Figure 6F,H). It was previously reported that the ubiquitin-proteasome pathway is one of the degradation pathways of the HSF1 protein [28]; thus, a deubiquitylation assay was performed to investigate whether USP14 could mediate the stabilization of the HSF1 protein by its deubiquitination with proteasome inhibitor MG132 treatment. The results showed that USP14 considerably reduced the HSF1 ubiquitination levels compared to the control group or Flag-USP14C114A group (Figure 6G), and the ubiquitination of HSF1 did not change in the presence of Flag-USP14C114A, indicating that USP14 was a functional DUB of HSF1. Taken together, these results revealed that USP14 stabilized the HSF1 protein through its deubiquitination.

### 3.7. HSF1 Correlated with USP14 Protein Levels in Human HNSCC Tissues

**Figure 7:**
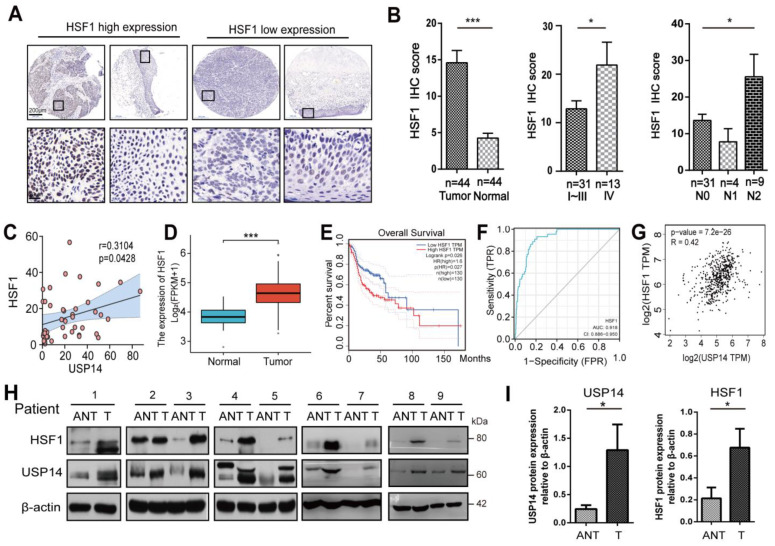
HSF1 is highly expressed in HNSCC tumors, and its expression is closely associated with USP14 expression. (**A**,**B**) Representative images of HSF1 IHC staining in HNSCC cancer and normal tissues derived from patients. An increased expression of HSF1 was shown in metastatic HNSCC cancer. (**C**) The relationship between USP14 and HSF1 was confirmed using Pearson’s correlation analysis (r = 0.3104, *p* < 0.05). (**D**) The box plot shows the HSF1 mRNA levels in normal tissues and HNSCC tissues from the TCGA database. (**E**) According to the median value of the HSF1 mRNA level, patients were classified into high and low groups. The Kaplan-Meier survival plot shows the overall survival of these two groups of patients. (**F**) The ROC curve of HSF1. The x-axis presents one specificity, and the *y*-axis presents sensitivity. (**G**) Pearson’s correlation analysis showed a correlation between USP14 and HSF1 in patients with HNSCC from the TCGA database. (**H**,**I**) The Western blot analysis of the expression of USP14 and HSF1 between tumor and adjacent normal tissues in 9 paired HNSCC patients. Data are analyzed by the mean ± SD at least three times. * Indicates *p* < 0.05, and *** indicates *p* < 0.0001.

To confirm the correlation between HSF1 expression and the HNSCC patients’ clinical characteristics, the IHC assays of 45 paired HNSCC cancer cases with paraffin sections available combined with their clinical characteristics were analyzed. As shown in Figure 7A, HSF1 expression was significantly elevated in each pathological stage of the HNSCC patients compared to the adjacent normal tissues, and the results indicated that the high expression of HSF1 was associated with the advanced tumor stage of patients and relatively high lymph node metastasis (Figure 7B). The relationships between the clinicopathological features of the HNSCC patients and HSF1 expression are shown in Supplementary Table S3. The Pearson correlation coefficient showed that the IHC scores of USP14 and HSF1 had a strong relationship in 45 pairs of HNSCC tissues (*p* = 0.0428) (Figure 7C). The expression of HSF1 was higher in tumor tissues than in normal tissues from the TCGA database (Figure 7D). The KM survival curve showed that HNSCC patients expressing a higher level of HSF1 had a poorer prognosis in the TCGA database (Figure 7E). The ROC analysis demonstrated that the prognostic accuracy of HSF1 expression in HNSCC patients reached 0.918, which was relatively high (Figure 7F). Pearson’s correlation analysis indicated that USP14 and HSF1 had a positive relationship in the TCGA database (Figure 7G). As shown in Figure 7H,I, Western blotting was applied to investigate the expression of USP14 and HSF1 in nine pairs of matched HNSCC tissue samples. The expression of USP14 and HSF1 was significantly higher in HNSCC tumor tissues (T) than in the corresponding adjacent normal tissues (ANT). In summary, these data indicated that the expression of HSF1 was significantly elevated in HNSCC tumor tissues compared to adjacent paired tissues, and its expression was correlated with lymph nodal metastasis, tumor grade, and USP14 expression in HNSCC patients.

### 3.8. USP14 Deficiency Represses Tumor Growth and Lung Metastasis In Vivo

**Figure 8:**
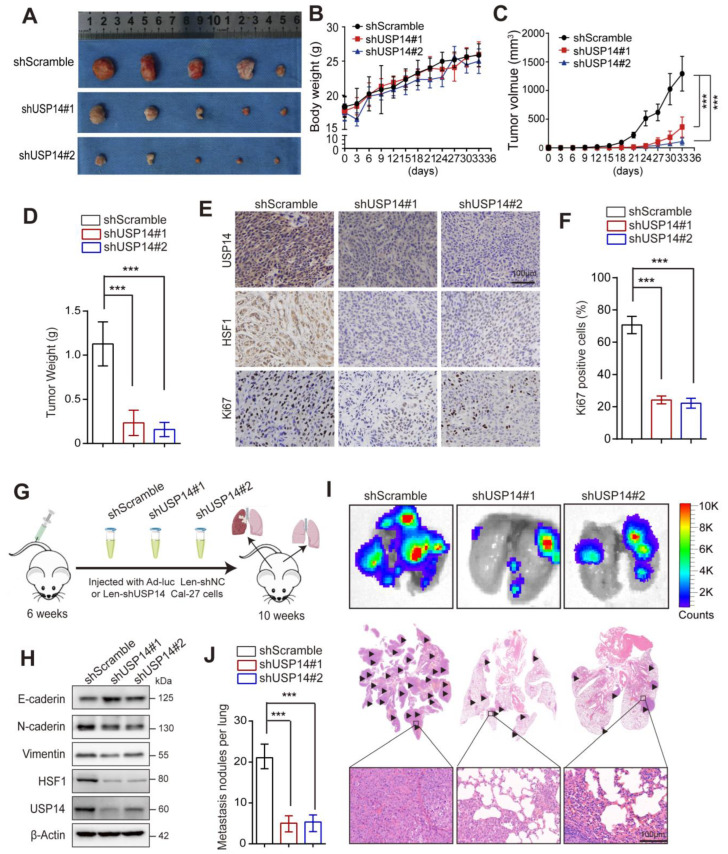
Knockdown of USP14 activity decreases the growth of HNSCC xenograft tumors and lung metastasis. (**A**) Comparison of the tumor size of shScramble, shUSP14#1, and shUSP14#2 cells. (**B**) The body weights of NOD/SCID mice were measured every 3 days, and the data from five separate biological replicates: tumor volume curve (**C**) and tumor weights (**D**) are shown. (**E**) Representative image showing the IHC analysis of USP14, HSF1, and Ki67 expression in HNSCC xenografts. (**F**) The percentage of Ki67-positive cells was quantified. Scale bar = 100 µm (*n* = 5). (**G**) A NOD/SCID mouse lung metastasis model was built. (**I**) Fluorescence imaging by live imager to observe lung metastasis in three groups. (**H**) The knockdown of USP14 in Cal-27 cells reduced the expression of EMT marker proteins. The protein expression of vimentin, E-cadherin, and N-cadherin was detected by Western blotting. (**J**) The number of lung metastatic nodules was analyzed by the Student’s *t*-test (*n* = 6). *** indicates *p* < 0.0001.

To further validate the biological role of USP14 in HNSCC, a subcutaneously implanted xenograft was utilized, and shScramble Cal-27 cells and shUSP14 Cal-27 cells were subcutaneously injected into NOD/SCID mice. It was found that the knockdown of USP14 decreased the growth of tumors (Figure 8A), although the weights of the mice in the shScramble group and the shUSP14 Cal-27 group showed no significant difference (Figure 8B). Consistently, the volume and weight of tumors in the USP14 depletion group were significantly reduced compared to those in the control group, indicating that the inhibition of USP14 could decrease the proliferation of HNSCC cells in vivo (Figure 8C,D). Immunohistochemistry (IHC) staining revealed that the protein levels of HSF1 and Ki67 decreased in the USP14-deficient groups (Figure 8E,F). Furthermore, lung metastasis models were established by the intravenous injection of fluorescently labeled Cal-27 cells to validate the influence of USP14 depletion on HNSCC cell lung metastasis in vivo (Figure 8G). The ex vivo lung and H&E staining of lung tissues showed that the number of metastases was significantly decreased in the Cal-27-shUSP14 group compared to the Cal-27-shScramble groups (Figure 8I,J). Western blotting showed that the expression levels of the EMT marker proteins N-cadherin and vimentin were downregulated and that E-cadherin was significantly upregulated in the USP14-deficient groups (Figure 8H). Collectively, these results indicated that USP14 played a pro-metastatic role in HNSCC progression.

### 3.9. Increased Expression of HSF1 Reverses the Inhibitory Effect of USP14 Depletion on the Proliferation and Metastasis of HNSCC in Mice

**Figure 9:**
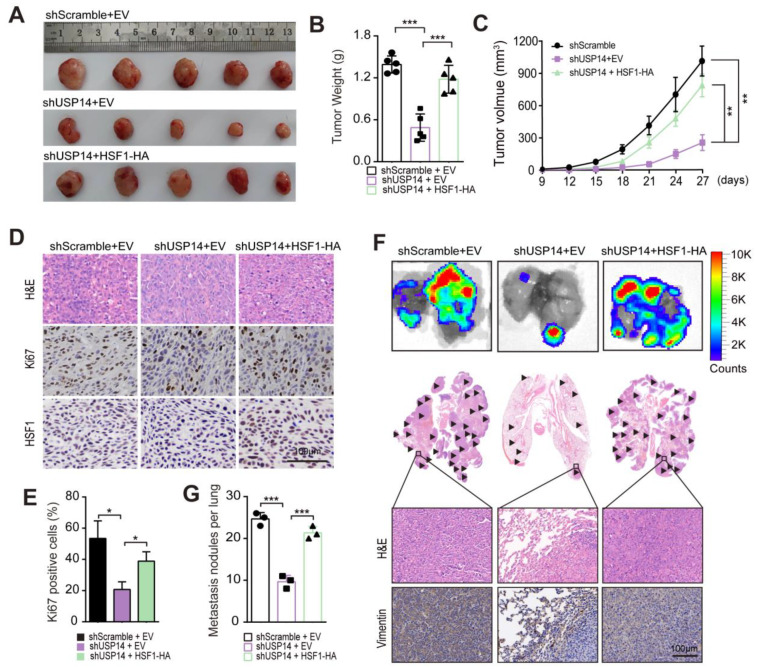
The upregulation of HSF1 expression counteracted the inhibitory effect of USP14 depletion in the growth and metastasis of HNSCC cells in vivo. (**A**) After a period of 27 days, ectopic HNSCC xenograft tumors were extracted and visually documented (*n* = 5). (**B**) The tumor weights were recorded (*n* = 5). (**C**) The volume of each mouse’s tumor was measured every three days, and growth curves depicting the changes in volume were generated. The data were presented as the mean ± SE values (*n* = 5) and analyzed using one-way ANOVA. (**D**,**E**,**G)** Staining and IHC assays (Ki-67 and HSF1) were performed on xenograft tumors derived from the three groups of cells. (**E**). The percentage of Ki67-positive cells were calculated. (**F**) Three groups were injected into the tails of NOD/SCID mice. After 21 days, the lungs of the mice were extracted and photographed, and a representative lung was shown. The black arrows indicate lung metastatic nodules. (**E**,**G**) Staining shows lung metastatic nodules (*n* = 6). (**F**) The number of lung metastatic nodules was calculated by the Student’s *t*-test (*n* = 6). * Indicates *p* < 0.05, ** indicates *p* < 0.001, and *** indicates *p* < 0.0001.

To further investigate the requirement of HSF1 for the oncogenic effects of USP14 on HNSCC cells, we transfected HSF1-HA into USP14-depleted cells through ectopic expression. Subsequently, NOD/SCID mice were subcutaneously implanted with three different types of cells: the control group (empty), the USP14 depletion group, and the HSF1 overexpression in USP14 depletion group. The mice were then divided into three groups based on the injected cells. After a 27-day period, it was observed that the ectopic expression of HSF1 reinstated the proliferation of HNSCC cells in vivo, which was previously diminished by the depletion of USP14 (Figure 9A–C). Similarly, the percentage of Ki67-positive cells reverted when HSF1 was overexpressed in the USP14 knockdown HNSCC samples (Figure 9D,E). Furthermore, the overexpression of HSF1 in HNSCC cells alleviated the inhibitory effects of USP14 knockdown on the metastatic ability in HNSCC cells (Figure 9 F,G), as evidenced by an increase in lung metastasis nodules in mice following HSF1 re-expression. Furthermore, in order to more accurately replicate human tumors, an orthotropic xenograft model was constructed to simulate the progression of tumor development in humans. These three types of Cal-27 cells were then injected into the tongues of nude mice; after 18 days, the orthotropic tumors formed. The observed gross appearance of the tongues, H&E staining, and percentage of Ki67-positive cells indicated that USP14-deficient cells exhibited slower tumor growth compared to the control groups; additionally, the overexpression of HSF1 restored the proliferation of HNSCC cells, which was previously impaired by USP14 depletion (Figure S4). In summary, these findings suggest that the overexpression of HSF1 plays a significant role in reversing the effects of USP14 depletion in HNSCC cells.

## 4. Discussion

Despite the obvious progression in the clinical therapeutic efficacy of surgical approaches, chemotherapy, and radiotherapy for HNSCC, it is still a disease with significant biodiversity and genomic heterogeneity. Detailed biological and molecular variability also promotes HNSCC’s highly heterogeneous nature. Taking into account the numerous functions of deubiquitinating modification observed in human tumors, DUB enzymes are important in the development of HNSCC; for example, USP9X [30] and USP22 [31] promote HNSC cell proliferation, and CYLD1 [32] and BPLF1 [33] accelerate HNSCC development by inhibiting the immune cell escape mechanism or by promoting the spread of viral infection. In this study, we systematically screened a pivotal driver gene, USP14, from the DUB library. A series of phenotype assays verified that USP14 was positively associated with cell proliferation and metastasis and decreased apoptosis in vitro, and a lung metastasis model and xenograft tumor model of NOD/SCID mice indicated that USP14 was positively associated with the tumor formation ability and lung metastasis in vivo. Regarding the clinical samples, IHC staining indicated that a high expression of USP14 was also associated with patients’ lymph node metastasis and tumor stage.

A malignant tumor is a microcosm of the pathological state caused by chronic toxic protein stress, which inevitably interferes with intracellular homeostasis in malignant transformation. Due to the special location of HNSCC, it is often exposed to high temperatures or biologically toxic food stimuli (such as smoking, drinking alcohol, or consuming betel nuts), which makes HSF1 an important transcription factor for the heat shock response under stress conditions to maintain protein homeostasis in HNSCC tumor cells. HSF1 acts as an oncogene and a potential diagnostic biomarker of tumors [34,35,36]. Previous studies have found that HSF1 functions in cells mainly through posttranslational modifications, such as phosphorylation [37], acetylation [38], sumoylation [39], and ubiquitination [40]. Existing evidence has shown that FBXW7α is a well-known ubiquitinating enemy of HSF1, and FBXW7 deficiency promotes HSF1 accumulation in melanoma cells and enhances their metastatic ability [40]. Currently, the only deubiquitinating enzyme of HSF1 reported is OTUB1, which promotes the proliferation and invasion of endometrial cells by promoting the protein stability of HSF1 through its deubiquitination [41], and they are colocalized in both the cytoplasm and nucleus. In our study, we found another new DUB of HSF1, USP14, that could change the HSF1 protein levels in a posttranslational manner. In our study, CHX, a deubiquitination assay, and the stability experiment CoIP also confirmed that USP14 could stabilize HSF1 by its deubiquitination.

USP14 is a deubiquitinating enzyme that is crucial to the survival and growth of eukaryotes. It is specifically combined with the 19S regulatory element of the proteasome and is catalytically activated. It was found that USP14 prefers ubiquity complex substrates carrying more than one ubiquitin modification or chain [42]. The catalytic activity of USP14 on the inhibition of degradation reflects its catalytic activity in the milliseconds before substrate degradation caused by the proteasome. The study found that, after heating, the polyubiquitination of HSF1 significantly increased, which is a sign of the rapid degradation of HSF1 by the proteasome after the removal of pressure [40]. Moreover, USP14 more easily targets substrates with polyubiquitin modification, so HSF1 may become a substrate for the deubiquitination of USP14 under objective conditions. In this study, we found that USP14 physically interacted with HSF1 and stabilized HSF1 by its deubiquitination. USP14 prolonged the half-life of the HSF1 protein. Furthermore, the overexpression of USP14 promoted the expression of HSF1 in a dose-dependent manner and increased the downstream transcriptional expression of HSF1 proteins, such as HSP60, HSP70, and HSP90s.

Numerous studies have shown that USP14 was highly expressed in different types of tumors, for example, in epithelial ovarian cancer, esophageal squamous cell carcinoma, colorectal cancer, lung adenocarcinoma, hepatocellular carcinoma cell and prostate cancer development, the expression level of USP14 protein is higher than that of normal tissue, and it is associated with poor prognosis in tumor patients [43,44,45,46,47,48]. USP14 also plays important roles in tumor death, for example, USP14 inhibits hepatocellular carcinoma apoptosis by stabilizing the GPX4 protein [49], and the inhibition of USP14 increases the degree of gastric cancer apoptosis, with an increased expression of cleaved caspase-3 and cleaved PARP [50]. In non-small cell lung cancer, USP14 is a regulator of major double-stranded break repair pathways under the ionizing radiation treatment to avoid lung tumor death [51]. In this research, we studied the impact of USP14 on the apoptosis of HNSCC, and we researched downstream of the USP14-HSF1 pathway. It has been suggested that HSF1 plays an important role in the death of tumor, and it has been reported that HSF1 can regulate the characteristic change of metabolic flux of glycolysis of cancer cells in vivo [52], so the inhibition of HSF1 activity leads slower proliferation and death of tumor cells. Interestingly, the activation of HSF1 has been associated with the upregulation of several antiapoptotic proteins, including Bcl-xL, MCL-1, and Bcl-2, and the survival rate, so the enhanced expression of these antiapoptotic proteins is shown to be mediated by the HSF 1 molecular chaperone HSP protein and BAG3 [53,54]. In addition, HSF1 activity inhibits the expression of SMAC, which is an indispensable protein in the mitochondrial pathway of apoptosis [55,56].

In a previous study, it was reported that the USP14 inhibitor bAP-15 could decrease the sensitivity of HNSCC cells to TNFα-mediated cell death, as well as radiation-induced cell death in HNSCC [57], but it did not precisely state the USP14 role in HNSCC development. Our research provided a new explanation for the mechanism of USP14 regulation in HNSCC and demonstrated, in detail, how USP14 promoted the HNSCC malignant phenotype. Collectively, our findings revealed that elevated USP14 is associated with lymph node metastasis and poor prognosis in HNSCC patients. USP14 promotes the proliferation and metastasis of HNSCC in vivo and in vitro. Our findings further identified that its new target, HSF1, and the downstream targets of HSF1 were also activated after USP14 was upregulated. Therefore, USP14 may serve as a novel biomarker, and our findings provide new ideas and approaches for the treatment of HNSCC.

Collectively, our findings revealed that elevated USP14 is associated with lymph node metastasis and poor prognosis in HNSCC patients. USP14 promotes the proliferation and metastasis of HNSCC in vivo and in vitro. Our findings further identified that its new target, HSF1, and the downstream targets of HSF1 were also activated after USP14 was upregulated. Therefore, USP14 may serve as a novel biomarker, and our findings provide new ideas and approaches for the treatment of HNSCC.

## 5. Conclusions

In conclusion, our study identifies USP14 as a novel deubiquitinating mediator that stabilizes HSF1 in HNSCC and enhances the downstream oncogenic response. The high abundance of USP14 and HSF1 in HNSCC indicated their key role as profound tumor-promoting factors, which is a previously unrecognized mechanism. Thus, our findings provide a new rationale and theoretical basis for novel treatment strategies for HNSCC.

## Data Availability

All data are available from the corresponding author for reasonable scientific reasons.

## Supplementary Materials

The following supporting information can be downloaded at https://www.mdpi.com/article/10.3390/cancers15174385/s1: Figure S1: The chosen process of DAGs. (A) Venn diagram showing overlap of the genes expressed in HNSCC patients among TCGA, GEO, and all DUBs. (B,C) Heat map and volcano plot showing 66 DAGs that were related to the overall survival of HNSCC patients after univariate Cox regression. Figure S2 Quantitative analysis of scratch healing assay to estimate the migration of TU686 (A), Cal-27 (B), and TU177 (C) cells. Figure S3: Identification of the downstream of USP14 regulated in HNSCC. (A) Heat map of differences in HSP-related proteins in TCGA database. (B) Gene set enrichment analysis (GSEA) revealed that adaptive thermogenesis pathway was enriched in patients with high expression of USP14 in GSE39366 database. Figure S4: Orthotropic xenograft showed overexpression of HSF1 restored the inhibitory effect of USP14 depletion in growth of HNSCC cells in vivo. (A). After 18 days, the orthotropic tumors on the tongue of nude mice were extracted and visually documented (*n* = 6). (B). H&E staining and IHC assays (Ki-67) were performed on orthotropic tumors derived from the three groups of cells. (C). The percentage of Ki67-positive cells were calculated (*** indicates *p* < 0.0001). Supplementary Table S1: The primers used in the study. Supplementary Table S2: The association between USP14 expression and the clinicopathological features of HNSCC patients. Supplementary Table S3: The relationships between the clinicopathological features of HNSCC patients and HSF1 expression. File S1: Original immunoblots.
