# Supplementary material for: USP14 Positively Modulates Head and Neck Squamous Carcinoma Tumorigenesis and Potentiates Heat Shock Pathway through HSF1 Stabilization
Source: Cancers (Basel). 2023 Sep 1;15(17):4385. doi: 10.3390/cancers15174385 (PMC10486363; doi:10.3390/cancers15174385)
Supplement: Supplementary file 1 [file cancers-15-04385-s001.zip › cancers-2539788-supplementary.pdf]

Supplementary Materials

Wang et al.

Supplemental Figures

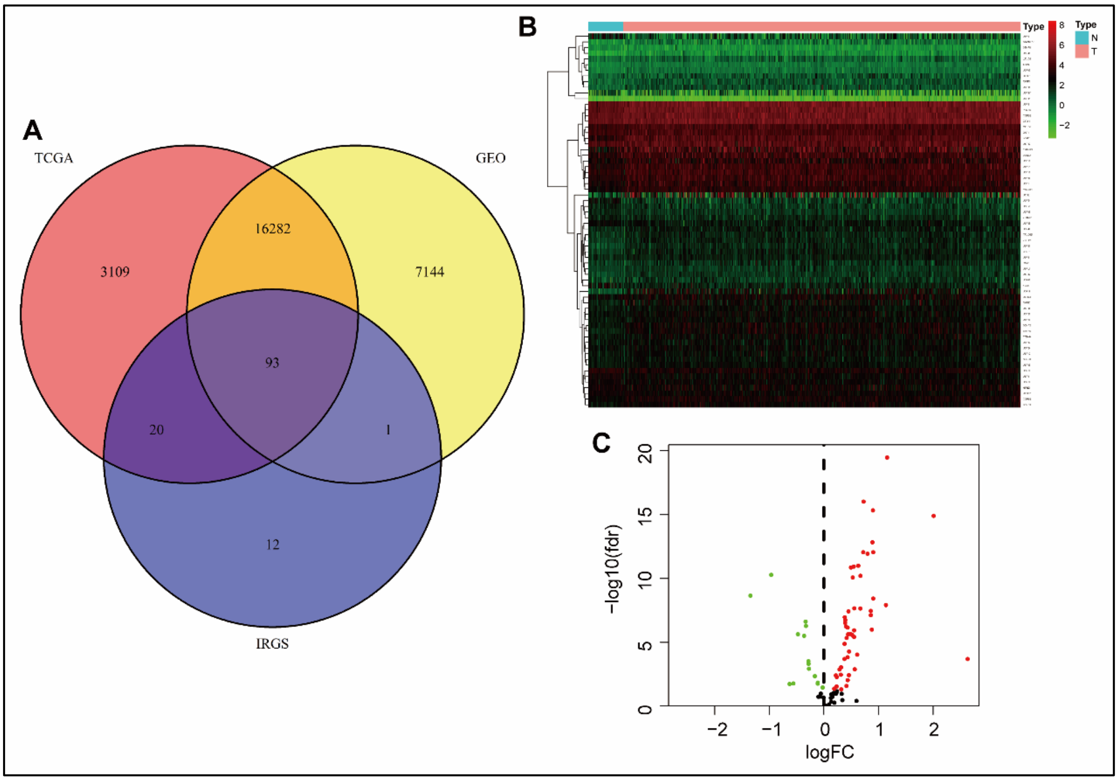

Figure S1 The chosen process of DAGs. **(A)** Venn diagram showing overlap of the genes expressed in HNSCC patients among TCGA, GEO and all DUBs; **(B-C)** Heat map and volcano plot showing 66 DAGs that were related to overall survival of HNSCC patients after univariate Cox regression;

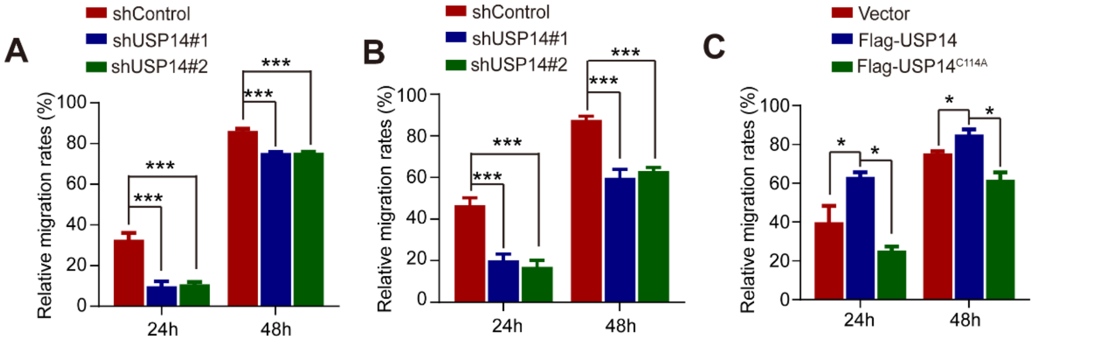

Figure S2 Quantitative analysis of scratch healing assay to estimate the migration of TU686(A) , Cal-27(B), and TU177 (C) cells.

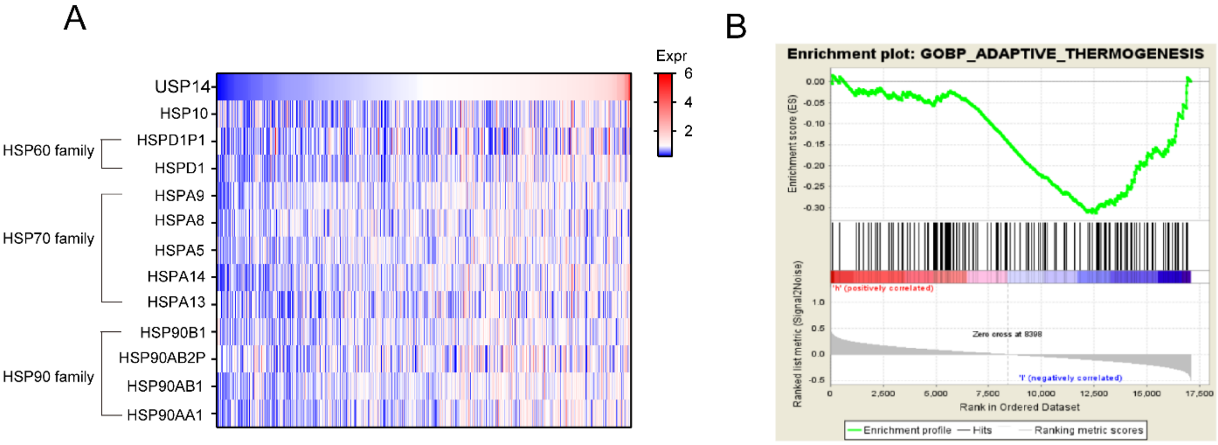

Figure S3 Identification of the downstream of USP14 regulated in HNSCC. (A) Heat map of differences in HSP-related proteins in TCGA database. (B) Gene set enrichment analysis (GSEA) revealed that adaptive thermogenesis pathway was enriched in patients with high expression of USP14 in GSE39366 database.

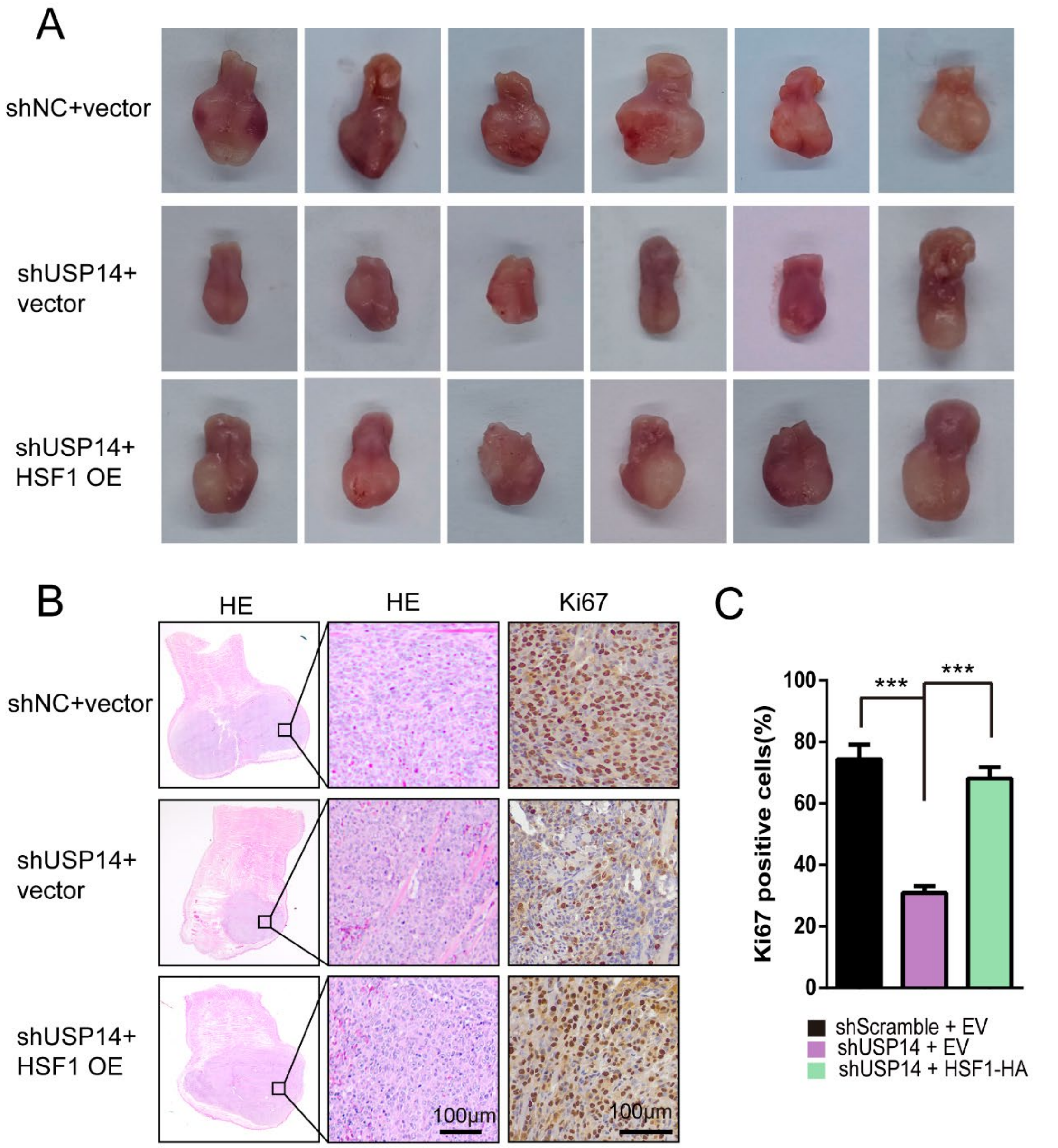

Figure S4 Orthotropic xenograft showed overexpression of HSF1 restored the inhibitory effect of USP14 depletion in growth of HNSCC cells in vivo. A. After 18 days, the orthotropic tumors on the tongue of nude mice were extracted and visually documented (n=6). B.H&E staining and IHC assays (Ki-67) were performed on orthotropic tumors derived from the three groups of cells. C. The percentage of Ki67-positive cells were calculated (* indicates $p<0.05$, ** indicates $p<0.001$ and *** indicates $p<0.0001$).

Supplemental Tables

Table S1. The primers used in the study.

gene	primer
USP14	GAGTTGGACCTTTCCAGA
USP14	TGCTTGCACAGATGTGA
HSF1	CCAGGAACTGGAAAGGCACTA
HSF1	CAGTGGTTGGTCCCAGTCTT
HSP60	GTGCTAATCCTGTTGGCATTC
HSP60	CCTGCGCGATTTCTTTCTTAC
HSP70	CGGCTCCGCTCTGAGATT
HSP70	CTGCGACAGTCCACTACCTT
HSP90	TCG CGG CAA AGA ACT CAC TT
HSP90	TTC TCA TCC TCG ACA CTG CTC T
β-actin	GAC TCC GGT CCT ATT TTG TGG
β-actin	GCT AGT TGG CAT CGT TTA TGG

Table S2. Association between USP14 expression and clinicopathological features of patients with HNSCC

		USP14 expression		
Clinicopathological features	n	Low	High	P-value
Age, years				0.8961
≥60	27	13	14	
< 60	16	9	7	
Sex				1
Male	42	21	21	
Female	1	1	0	
Histological grade				0.3598
G1	28	14	14	
G2+G3	15	8	7	
T				0.5116
T1	12	9	3	
T2	19	6	13	
T3	7	4	3	
T4	5	3	2	
M				1
M0	43	22	21	
Lymph node metastasis				**0.0039**
N0	30	20	10	
N1	4	2	2	
N2	9	0	9	
Cancer stage				**0.0438**
I+II+III	30	19	11	
IV	13	3	10	
Infiltration				0.3119

submucosa	11	8	3	
superficial muscularis	9	3	6	
deep muscularis	12	6	6	
whole layer	11	4	7	
Tissues type				**0.0012**
Cancer	43	22.98±0.492		
Adjacent normal tissues	43	7.349±0.268		

Table S3. Association between HSF1 expression and clinicopathological features of patients with HNSCC

		HSF1 expression		
Clinicopathological features	n	Low	High	P-value
Age, years				0.5617
≥60	28	13	15	
< 60	16	9	7	
Sex				1
Male	42	21	20	
Female	1	1	0	
Histological grade				0.1395
G1	29	12	17	
G2+G3	15	9	6	
T stage				0.6638
T1	13	6	7	
T2	19	7	12	
T3	7	6	1	
T4	5	3	2	
M				1
M0	44	44	0	
N stage				**0.0146**
N0	31	16	15	
N1	4	3	1	
N2	9	3	6	
cancer stage				**0.0276**
I+II+III	31	16	15	
IV	13	6	7	
Infiltration				0.6539
submucosa	11	6	5	
superficial muscularis	10	3	7	
deep muscularis	12	7	5	
whole layer	11	6	5	
Tissues type				**< 0.0001**
Cancer	44	15.549±0.285		
Adjacent normal tissues	44	4.252±0.101		
