# Supplementary material for: USP14 Positively Modulates Head and Neck Squamous Carcinoma Tumorigenesis and Potentiates Heat Shock Pathway through HSF1 Stabilization
Source: Cancers (Basel). 2023 Sep 1;15(17):4385. doi: 10.3390/cancers15174385 (PMC10486363; doi:10.3390/cancers15174385)

Full unedited gel for Figure 3A

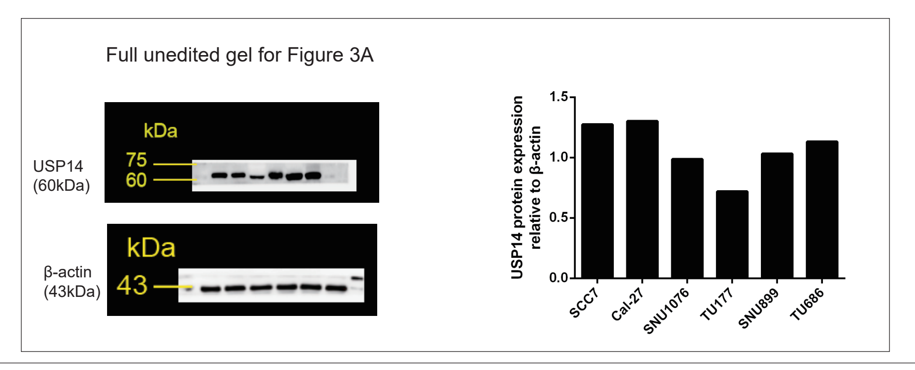


Full unedited gel for Figure 3B

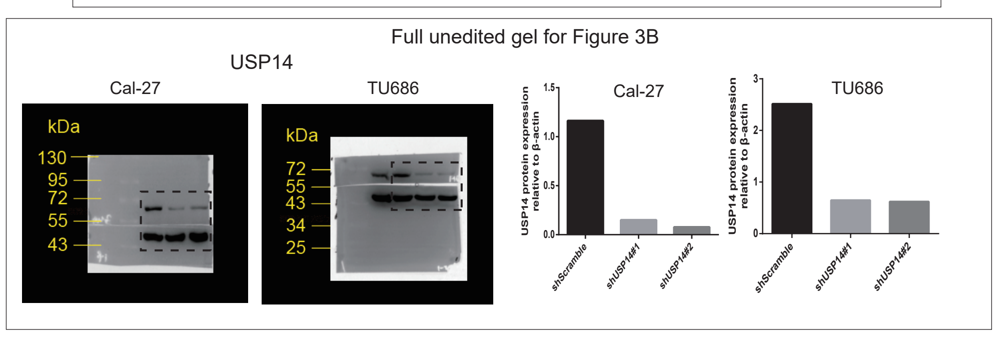


Full unedited gel for Figure 4A

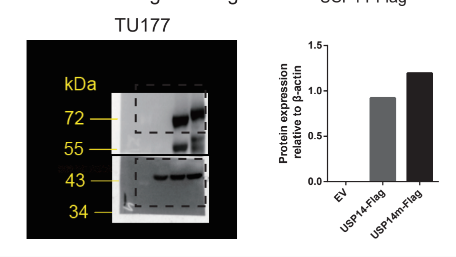


Full unedited gel for Figure 5D

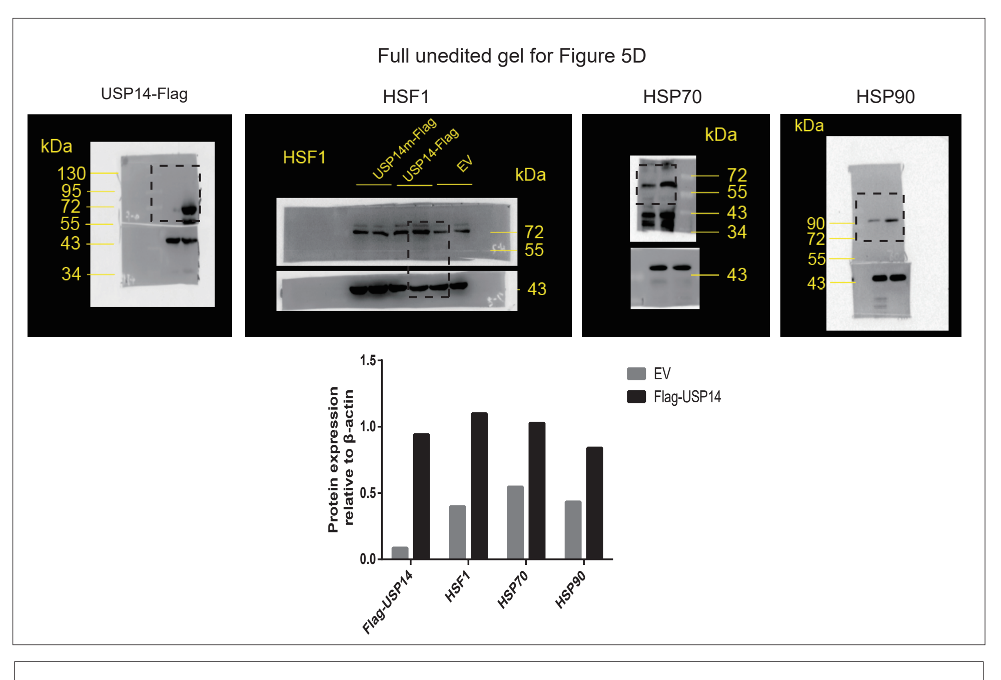


Full unedited gel for Figure 5E

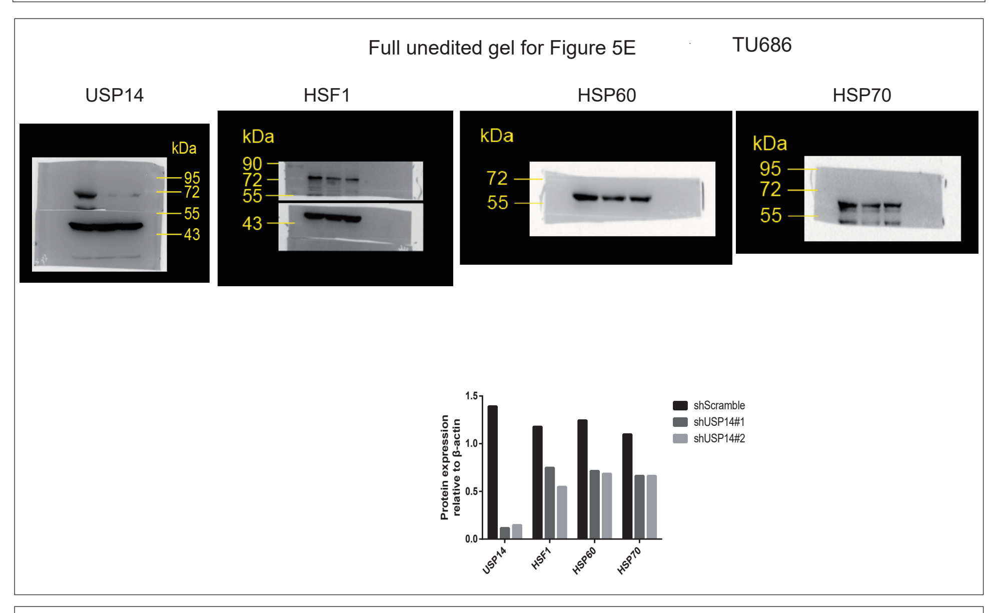


Full unedited gel for Figure 5F

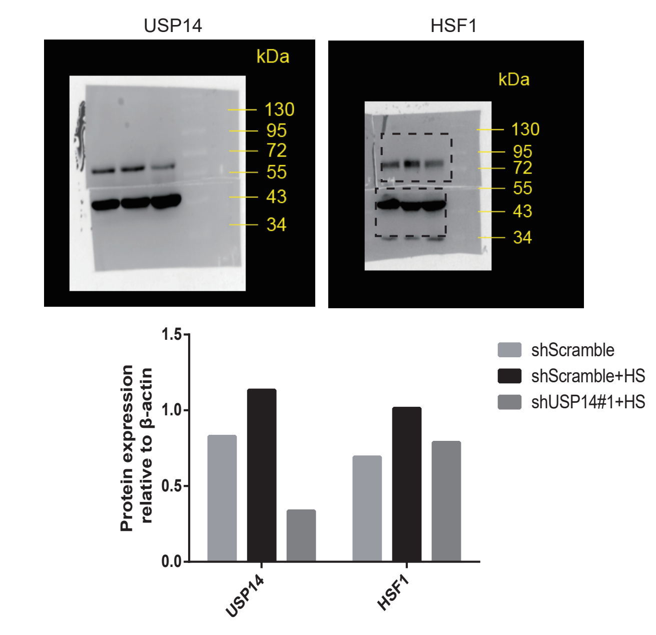


Full unedited gel for Figure 5G

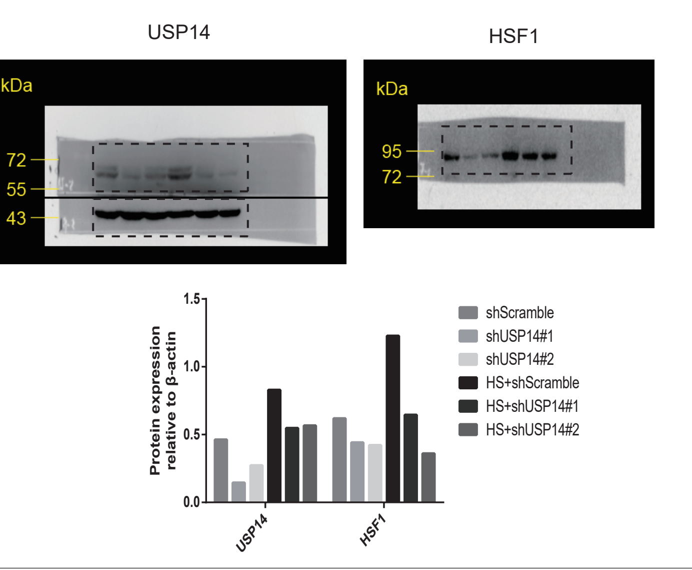


Full unedited gel for Figure 6A

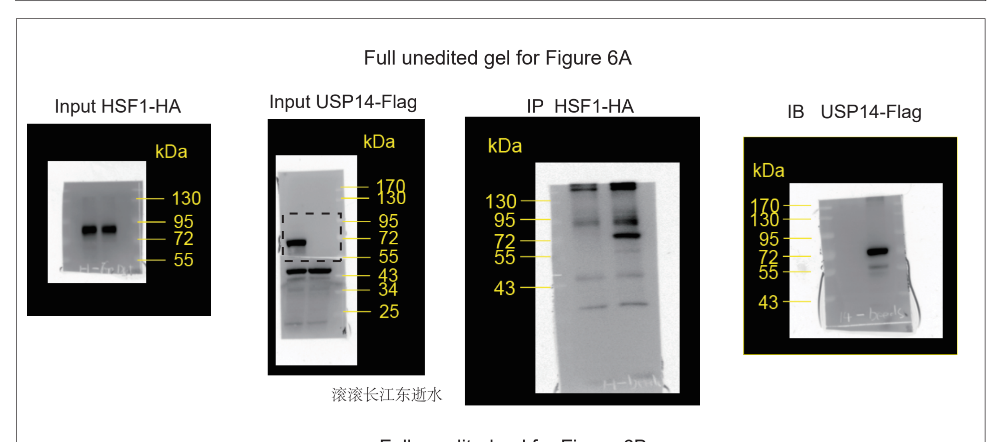


Full unedited gel for Figure 6B

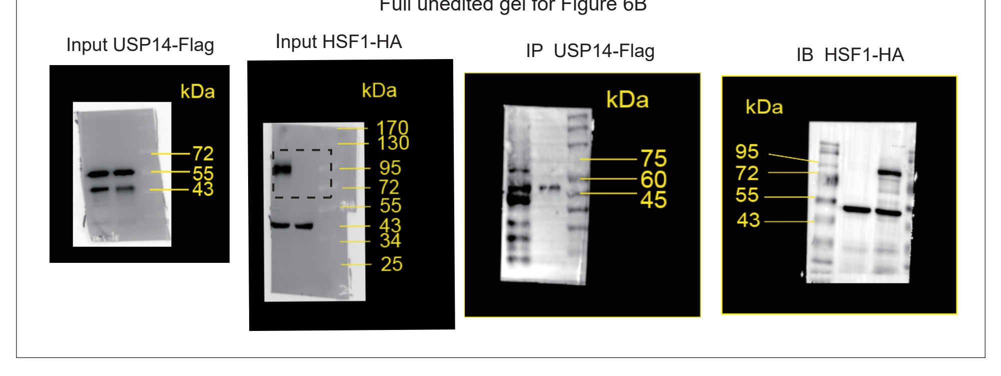


Full unedited gel for Figure 6C

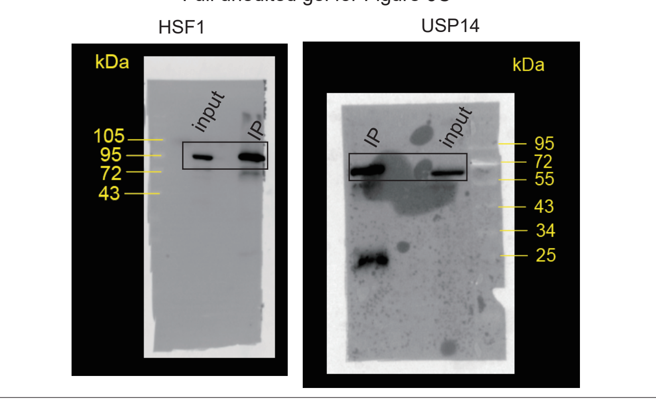


Full unedited gel for Figure 6E

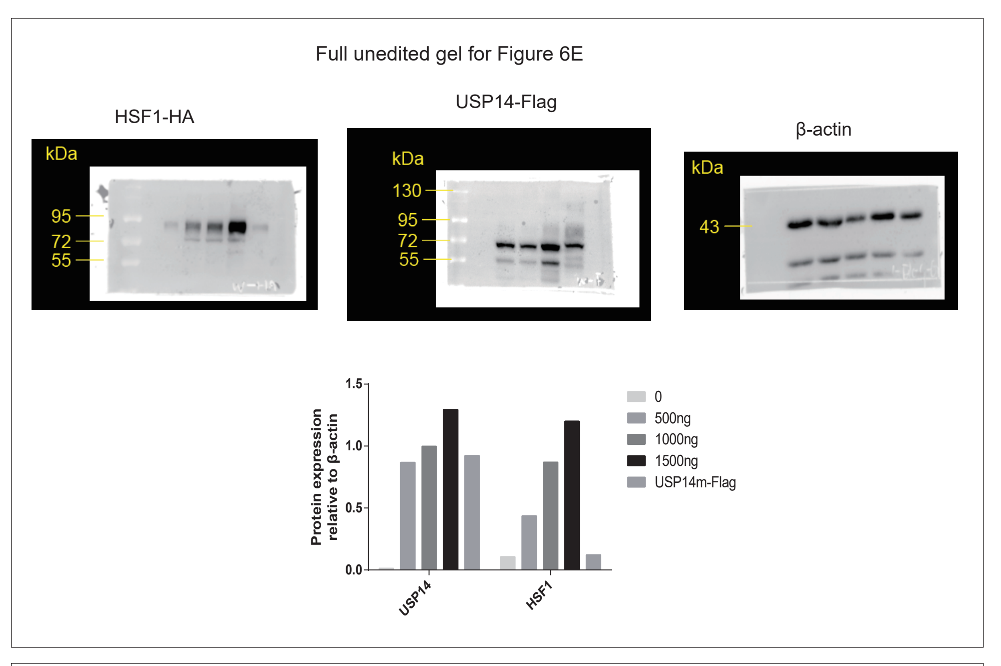


Full unedited gel for Figure 6F

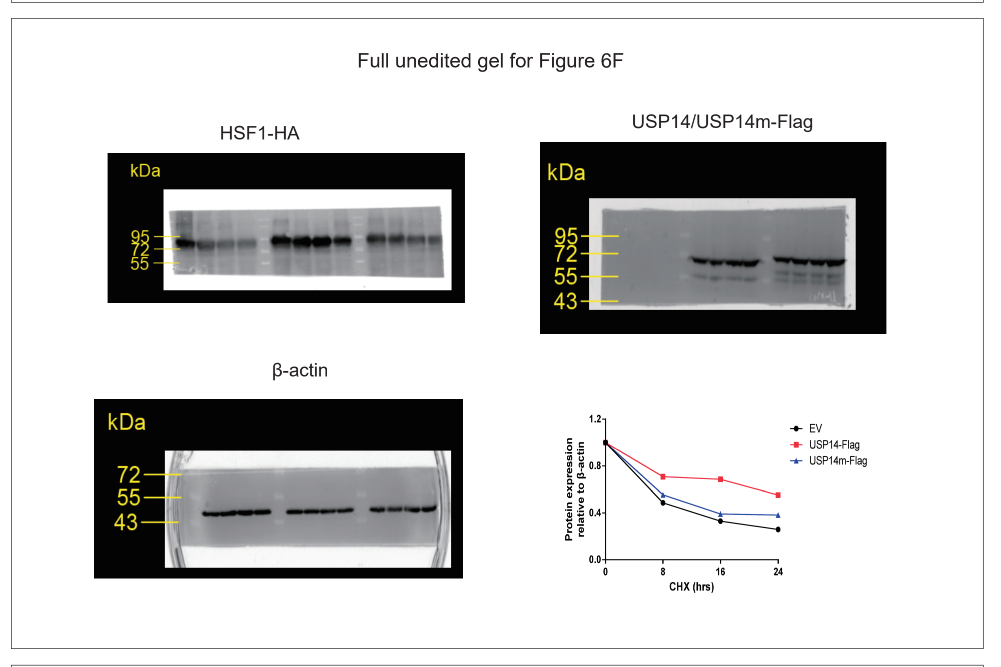


Full unedited gel for Figure 6G

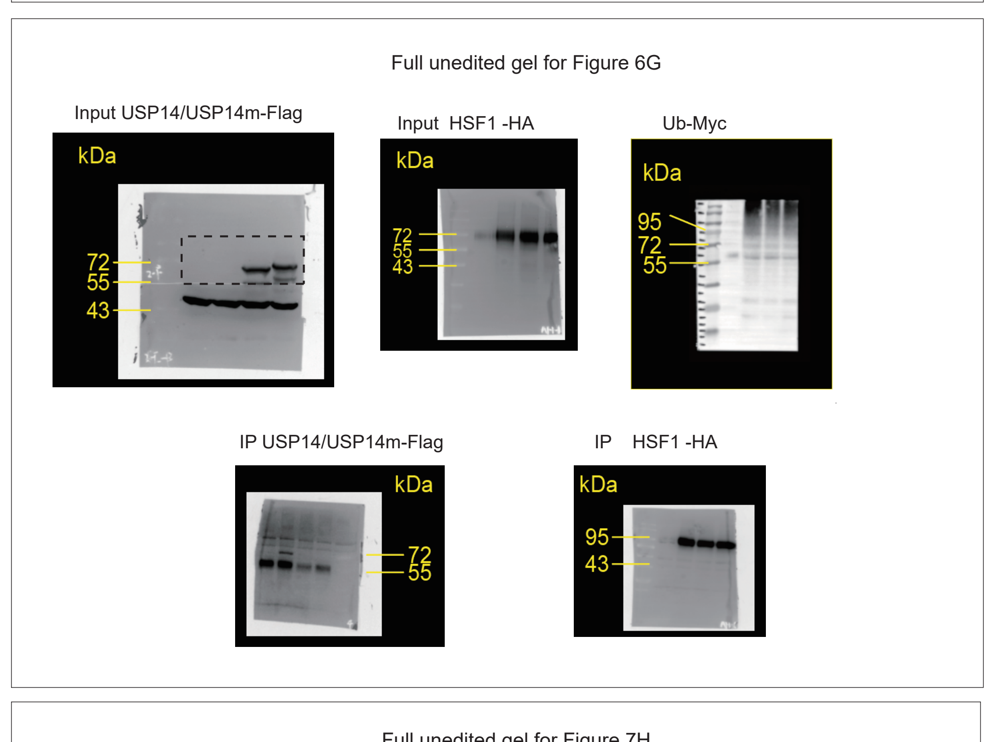


Full unedited gel for Figure 7H

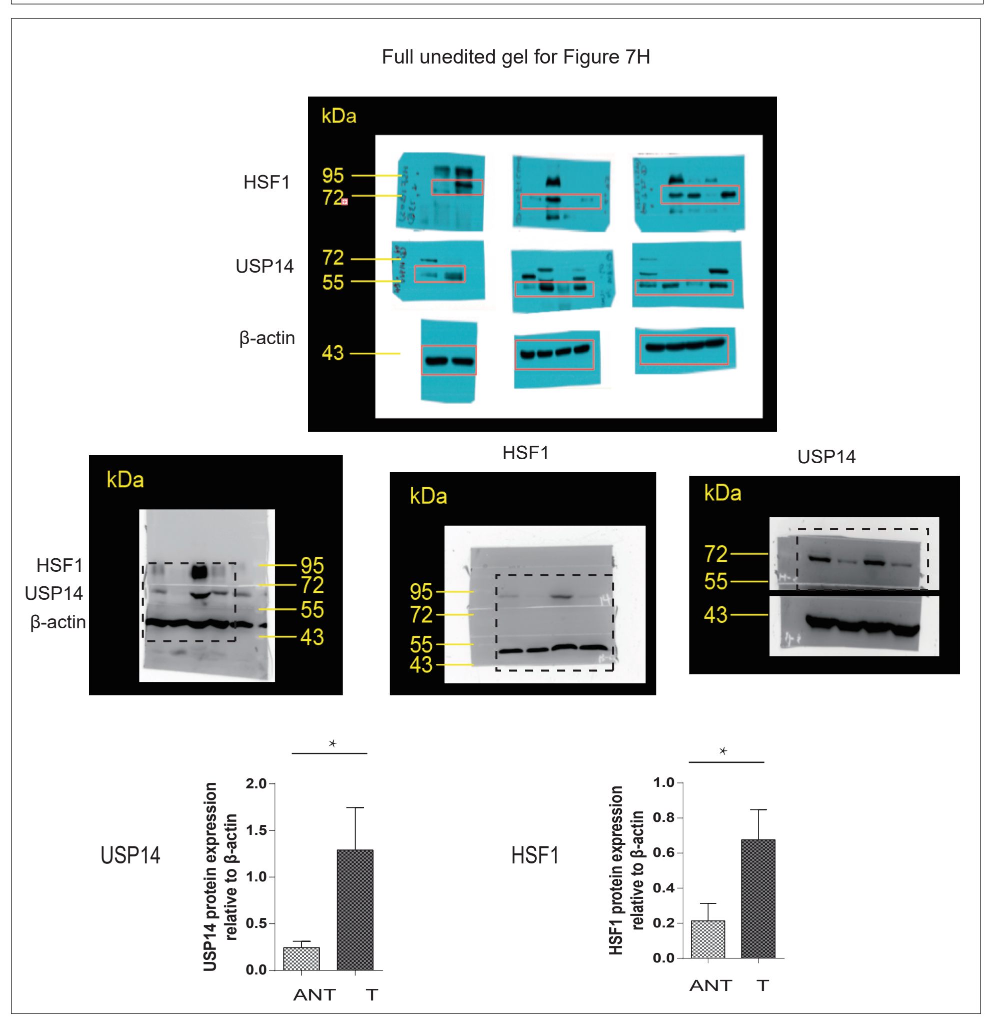


Full unedited gel for Figure 8H

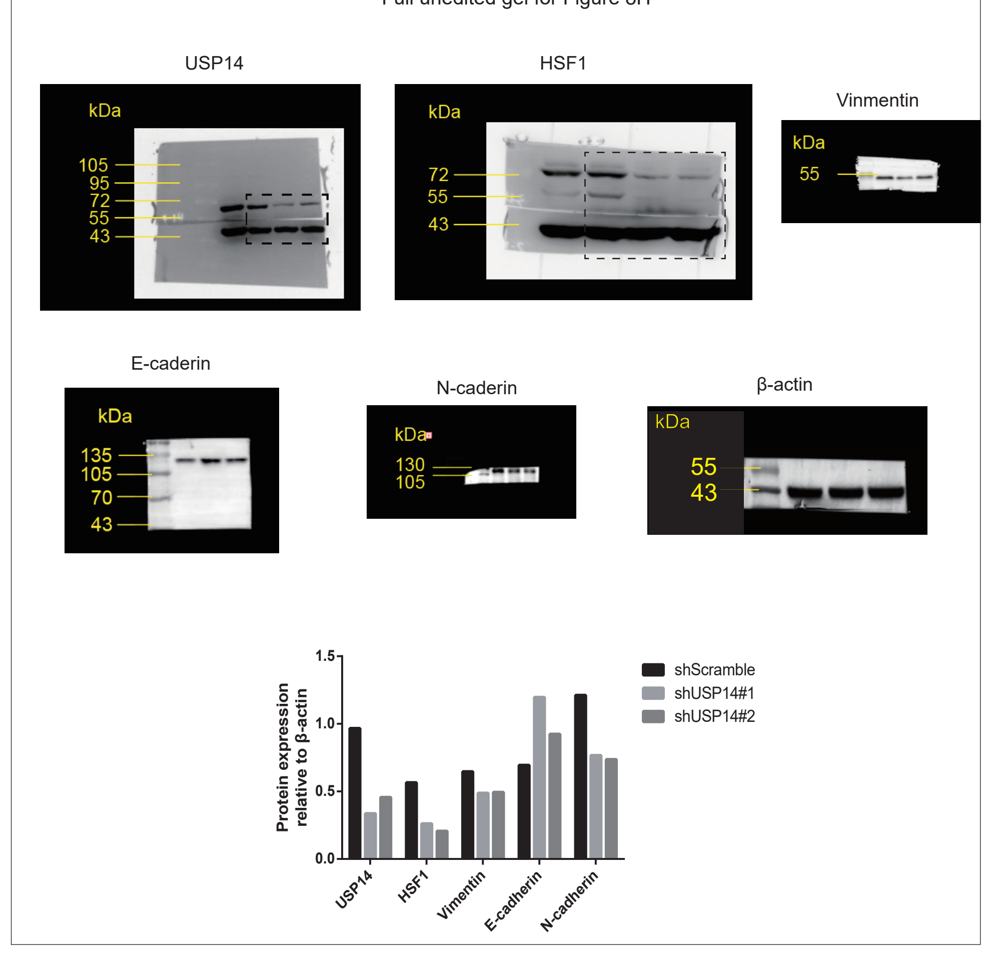

Supplement: Supplementary file 1 [file cancers-2539788_original images.pdf › cancers-2539788_original images.pdf]
